# The pesticidal Cry6Aa toxin from *Bacillus thuringiensis* is structurally similar to HlyE-family alpha pore-forming toxins

**DOI:** 10.1186/s12915-016-0295-9

**Published:** 2016-08-30

**Authors:** Alexey Dementiev, Jason Board, Anand Sitaram, Timothy Hey, Matthew S. Kelker, Xiaoping Xu, Yan Hu, Cristian Vidal-Quist, Vimbai Chikwana, Samantha Griffin, David McCaskill, Nick X. Wang, Shao-Ching Hung, Michael K. Chan, Marianne M. Lee, Jessica Hughes, Alice Wegener, Raffi V. Aroian, Kenneth E. Narva, Colin Berry

**Affiliations:** 1 Shamrock Structures LLC, Woodridge, IL USA; 2 Cardiff School of Biosciences, Cardiff University, Park Place, Cardiff, CF15 8FA UK; 3 University of Massachusetts Medical School, 373 Plantation Street, Worcester, MA 01605-2377 USA; 4 Dow AgroSciences, LLC, Indianapolis, IN USA; 5 6125 Londonberrie Ct., Midland, MI 48640 USA; 6 School of Life Sciences and Center of Novel Biomaterials, The Chinese University of Hong Kong, Shatin, HK SAR China; 7 Present address: Indiana State Department of Health Laboratories, Indianapolis, IN USA; 8 Present address: Laboratorio de Interacción Planta-Insecto, Departamento de Biología Medioambiental, Centro de Investigaciones Biológicas – CSIC, Madrid, Spain; 9 Present address: Antimicrobial Reference Laboratory, Southmead Hospital, Westbury-on-Trym, Bristol, BS10 5NB UK; 10 Present address: Xylogenics, LLC, Indianapolis, IN USA

**Keywords:** *Bacillus thuringiensis*, Cry6, Insecticidal toxin, Hemolysin

## Abstract

**Background:**

The Cry6 family of proteins from *Bacillus thuringiensis* represents a group of powerful toxins with great potential for use in the control of coleopteran insects and of nematode parasites of importance to agriculture. These proteins are unrelated to other insecticidal toxins at the level of their primary sequences and the structure and function of these proteins has been poorly studied to date. This has inhibited our understanding of these toxins and their mode of action, along with our ability to manipulate the proteins to alter their activity to our advantage. To increase our understanding of their mode of action and to facilitate further development of these proteins we have determined the structure of Cry6Aa in protoxin and trypsin-activated forms and demonstrated a pore-forming mechanism of action.

**Results:**

The two forms of the toxin were resolved to 2.7 Å and 2.0 Å respectively and showed very similar structures. Cry6Aa shows structural homology to a known class of pore-forming toxins including hemolysin E from *Escherichia coli* and two *Bacillus cereus* proteins: the hemolytic toxin HblB and the NheA component of the non-hemolytic toxin (pfam05791). Cry6Aa also shows atypical features compared to other members of this family, including internal repeat sequences and small loop regions within major alpha helices. Trypsin processing was found to result in the loss of some internal sequences while the C-terminal region remains disulfide-linked to the main core of the toxin. Based on the structural similarity of Cry6Aa to other toxins, the mechanism of action of the toxin was probed and its ability to form pores in vivo in *Caenorhabditis elegans* was demonstrated. A non-toxic mutant was also produced, consistent with the proposed pore-forming mode of action.

**Conclusions:**

Cry6 proteins are members of the alpha helical pore-forming toxins – a structural class not previously recognized among the Cry toxins of *B. thuringiensis* and representing a new paradigm for nematocidal and insecticidal proteins. Elucidation of both the structure and the pore-forming mechanism of action of Cry6Aa now opens the way to more detailed analysis of toxin specificity and the development of new toxin variants with novel activities.

**Electronic supplementary material:**

The online version of this article (doi:10.1186/s12915-016-0295-9) contains supplementary material, which is available to authorized users.

## Background

*Bacillus thuringiensis* strains produce a range of toxins active against invertebrates with enormous potential for use in the control of pests of importance in agriculture and health [1]. During sporulation, strains synthesize cytolytic (Cyt) toxins and/or crystal (Cry) protoxins and deposit them as parasporal inclusion bodies. Of the 74 major subclasses of Cry protoxin currently recognized (see http://www.lifesci.susx.ac.uk/home/Neil_Crickmore/Bt/) [2], most belong to a large family of related sequences that can be proteolytically processed to yield active toxins with a three-domain fold as first seen for Cry3Aa [3]. However, the Cry nomenclature is not limited to these proteins and includes several distinct and unrelated lineages. While much is known of the structure and function of the three-domain toxins [4] and the structure of a protoxin form has recently been published [5], very little is known of the non-three-domain Cry proteins. Some of these proteins share sequence homology to other known toxins; for example, Cry35 and Cry36 are related to the Bin and Cry49 toxins of *Lysinibacillus sphaericus* [6] and may share a proposed mechanism of action involving pore formation [7] and/or apoptosis [8]. The structures of the ß-sheet-rich toxins Cry45 (Parasporin4) [9], Cry46 (Parasporin 2) [10], and recently Cry51 [11] and the Cry34/Cry35 binary toxin [12] have been published but for many non-three-domain Cry proteins, neither structural data nor information on mechanism of action are available. One such Cry protein is Cry6Aa [13], a protein with activity against Coleoptera such as the Western Corn Rootworm *Diabrotica virgifera virgifera* [14] and a range of nematodes, including both free-living (*Caenorhabditis elegans* and *Panagrellus redivivus*) and plant pathogenic (*Heterodera glycines* and *Meloidogyne incognita*) species [15–18] that cause large-scale losses to agriculture [19].

Previous studies have shown by deletion of Cry6Aa sequences that 10 amino acids can be deleted from the N-terminus and 93 residues can be removed from the C-terminus, leaving a core region of ~43 kDa that retains activity against nematode targets [15]. Cry6B is a protein that is closely related to Cry6A but lacks 88 amino acids that are seen at the C-terminus of the latter protein. Cry6B is reported to be active against the coleopteran lucerne weevil *Hypera postica* [20] but showed little or no activity against a range of nematode targets [15]. The absence of further data on the structure and function of these proteins has limited our ability to understand their activity against target invertebrates. As a result, development and exploitation of the toxins in the control of agricultural pest insects and nematodes pathogenic to plants and animals may be limited. This, in turn, inhibits their use to supplement the current chemotherapeutic approaches to nematicidal treatments that are very toxic and are being phased out [21]. In this study we applied both crystallographic techniques and state of the art ab initio modeling to probe the structure of Cry6Aa in protoxin and trypsin-cleaved forms. The structures obtained are novel among invertebrate-active toxins and are consistent with Cry6Aa acting as a pore-forming toxin. We demonstrated pore formation in vivo and used our predictions to construct a mutant expected to show compromised toxicity.

## Methods

### Bacterial strains

*B. thuringiensis* strain BMB171 containing the pHT304-derived plasmid encoding the Cry6Aa2 protein [22] was kindly provided by Dr Ming Sun, Huazhong Agricultural University, Wuhan, China. This strain was grown to greater than 98 % autolysis (as judged by phase contrast microscopy) in Embrapa medium [23]. Spores were harvested and crystals of Cry6Aa were isolated and purified by discontinuous sucrose density gradient ultracentrifugation according to previously described methods [6]. Cry6Aa protein crystals purified by this method were solubilized at 37 °C with shaking at either alkaline pH by incubation in 50 mM NaOH (final pH 12.7) or at acidic pH by the addition of 100 mM sodium citrate buffer pH 3.0.

### Toxin production in *Escherichia coli*

The *cry6Aa* gene was amplified from the above *B. thuringiensis* strain by 30 cycles of PCR with a 60 °C annealing temperature, using forward (5′-GTCGAC*GAATTC*AATGATTATTGATAGTAAAACGAC-3′) and reverse (5′-GTCGAC*GAATTC*ACAATTAATTATTATACCAATCCG-3′) primers, incorporating *Eco*RI restriction sites (shown in italics). The amplicon was cloned into the pGEM-T vector (Promega, Southampton, UK) and transformed into *E. coli* DH5α for the selection of a single colony, plasmid isolation, and sequencing to confirm the cloning of the gene with no mutations. The gene was subsequently cleaved from this plasmid with *Eco*RI and re-cloned into the *Eco*RI site of the pET28b expression vector to allow production of recombinant Cry6Aa protein with an N-terminal His-tag. A mutation of the *cry6Aa* gene to change Leu259 to Asp was introduced using the Phusion site-directed mutagenesis kit (ThermoFisher Scientific, Loughborough, UK) according to the manufacturer’s instructions by PCR (98 °C for 30 s, followed by 25 cycles of 98 °C for 10 s, 57 °C for 30 s, and 72 °C for 200 s) using the pET28b clone as template and the following 5′-phosphorylated oligonucleotides: 5′-*GAC*TTAGGATTTGTTGTTTATGAAATTC-3′ (TTG to GAC mutation shown in italics), 5′-TGGTCCTAATAGAAAACTATATTC-3′. Following ligation of the PCR product and transformation of *E. coli* DH5α, a colony was picked and the plasmid sequence was verified to ensure incorporation of the desired mutation and no other changes.

For recombinant production of Cry6Aa proteins in *E. coli*, the pET28b plasmids were transformed into the Shuffle T7 Express LysY *E. coli* strain (New England Biolabs, Hitchen, UK) and isolated colonies were selected. Transformants were grown in lysogeny broth (LB) medium in the presence of 50 μg/ml kanamycin at 37 °C with shaking overnight before the addition of isopropyl ß-D-thiogalactopyranoside (IPTG) to a final concentration of 1 mM and growth for a further 3 h. Expression was verified by western blotting using monoclonal anti-polyhistidine antibody (Sigma-Aldrich, Gillingham, UK: Catalog code H1029 RRID:AB_260015) as the primary antibody and rabbit anti-mouse alkaline phosphatase conjugate antibody (Sigma-Aldrich: Catalog code A4312, batch number 095 K4758) as the secondary antibody to allow visualization of the proteins using the AP Conjugate Substrate Kit (Bio-Rad, Hemel Hempstead, UK).

For in vivo pore formation assays, a distinct clone of the *cry6Aa* gene in the *Bam*HI and *Pst*I sites of the vector pQE9 was produced (adding an N-terminal His-tag and a seven amino-acid extension to the C-terminus, encoded by the vector). An equivalent clone in pQE9 containing the *cry5Ba* gene as a positive control for in vivo pore formation has been described previously [24].

### Cry6 production in *Pseudomonas fluorescens*

For inclusion body (IB) preparation, *Pseudomonas fluorescens*-derived cell paste expressing full-length, wild-type Cry6Aa1 (from Dow AgroSciences [GenBank:AAA22357]) was transferred from −80 °C storage to 4 °C and resuspended at 20 % w/v in cold lysis buffer [50 mM TrisHCl, 200 mM NaCl, 10 % glycerol, 0.5 % Triton X-100, 20 mM EDTA, 1 mM Tris(2-carboxyethyl)phosphine (TCEP), pH 7.5] and mixed thoroughly with a handheld homogenizer. The suspension was then passed through a Microfluidizer (Microfluidics M-110EH) twice at 16,000 psi then centrifuged (SLC-6000 rotor, 14,000 g for 40 min at 4 °C). The supernatant was discarded. The IB pellet was fully resuspended by homogenization in 10 % w/v room temperature lysis buffer with 0.4 g/l lysozyme (L-6876; Sigma-Aldrich). The suspension was incubated at 30 °C for 30 min, with brief homogenization every 10 min. The IBs were then centrifuged (SLC-6000 rotor, conditions as above) and the supernatant was discarded. The pellet was resuspended for a final time in 10 % w/v cold lysis buffer using the homogenizer and then centrifuged (SLC-6000 rotor, conditions as above) and the supernatant discarded. The IB pellet was resuspended in 10 % w/v cold lysis buffer without Triton X-100 (50 mM TrisHCl, 200 mM NaCl, 10 % glycerol, 20 mM EDTA, 1 mM TCEP, pH 7.5) using the homogenizer and then centrifuged (SLC-6000 rotor, conditions as above) and the supernatant discarded. This step was repeated twice. The IBs were resuspended to 30 % (w/v) in 10 mM EDTA, pH 8.0, and then separated into 1.5 ml aliquots and frozen at −80 °C until needed. A separate aliquot of the IB preparation was stored without suspension in 10 mM EDTA.

#### Purification of full-length Cry6Aa

About 500 mg of *Pseudomonas*-derived IB paste was thawed at 4 °C, and centrifuged at 23,000 g for 25 min at 4 °C. The supernatant was removed and the pellet was solubilized in 30 ml of 100 mM CAPS, pH 11.0, and the suspension was gently rocked for 2 h at room temperature to solubilize the Cry6Aa. After solubilization, the mix was centrifuged at 23,000 g for 25 min at 4 °C. The supernatant was dialyzed against 25 mM CAPS, pH 10.0, overnight.

The buffer exchanged Cry6Aa sample was then filtered through a 0.22 μm syringe filter and applied to a Source 15Q 16/6 ion exchange column at a flow rate of 5 ml/min. The column was pre-equilibrated with buffer A (25 mM CAPS, pH 10). The protein was eluted with a step gradient of 20 %, 30 %, 40 %, and 50 % buffer B (buffer A + 1 M NaCl). Each elution was ~50 ml. Fractions were collected from each elution based on UV absorbance and analyzed by sodium dodecyl sulfate polyacrylamide gel electrophoresis (SDS-PAGE). Full-length Cry6Aa (475 amino acids) was in the 20 % B fractions, which were pooled and concentrated to ~30 ml using a centrifugal filter device with 10 kDa molecular weight cut-off membrane (Millipore, Cal# UFC901024).

The concentrated sample was further purified by size exclusion chromatography. For each run, 4.0 ml of the sample was applied over a Superdex 75 26/90 gel filtration column pre-equilibrated in 25 mM CAPS, pH 10, and 50 mM NaCl buffer, at a flow rate of 2.5 ml/min. Two peaks were observed (monitored by absorbance at 280 nm). The first peak, eluted at the void volume, contained Cry6Aa dimers (judged by non-denaturing and denaturing SDS-PAGE, with only monomers of Cry6Aa appearing in the latter case). The fractions from peak 2 contained predominantly monomer and were pooled and used in crystallization experiments.

#### Purification of Cry6Aa trypsin-resistant core

Eight 2 ml tubes of IB suspension of full-length Cry6Aa were thawed and extracted in 80 ml final volume (1:5 dilution) of 10 mM CAPS, pH 11.0. The measured pH of 9.1 was then adjusted to 11.0 with NaOH. Freshly prepared trypsin [2 ml of 25 mg/ml trypsin solution (Sigma, T1426-1G TLCK treated] in 1 mM HCl, 5 mM CaCl_2_) was added, the pH was maintained at 11.0, and the mixture was stirred overnight at 4 °C.

The digestion was applied to a Source 15Q 16/10 column pre-equilibrated in buffer C (25 mM CAPS, pH 11.0) at a flow rate of 10 ml/min and eluted with a gradient of buffer C + 1 M NaCl over 75 min. Trypsin-processed Cry6Aa eluted as a single large peak, which was concentrated from 100 ml to 10 ml using 15 ml Amicon 10,000 MWCO spin concentrators in a JA-12 rotor at 4 °C and 5000 g.

The concentrated ion exchange sample was applied to a Superdex 75 26/90 gel filtration column pre-equilibrated in 25 mM CAPS, pH 11.0, and 50 mM NaCl. The sample was eluted using a 2.5 ml/minute flow rate. The fractions corresponding to the large Cry6Aa core peak were pooled (80 ml at 2.05 mg/ml) for crystallization trials.

### Secondary structural analysis

Circular dichroism (CD) spectra were recorded using a JASCO J-815 CD spectrometer equipped with an MPTC-490S 6-cell Peltier Thermostatted cell holder. The instrument was calibrated with (1S)-(+)-10-camphorsulfonic acid. Measurements were carried out in a 1 mm path-length quartz cuvette under a constant nitrogen stream and at room temperature (25 °C). Data were collected in continuous scan mode from 260 to 185 nm every 0.5 nm at 50 nm/min and with 2 s averaging per point. Each spectrum is the average of five accumulations and a bandwidth of 1 nm. The concentration of Cry6Aa1 protein in 10 mM phosphate pH 3.0 was 0.09 mg/ml, in 10 mM phosphate pH 8.0 was 0.09 mg/ml, and in 10 mM CAPS pH 11.0 was 0.085 mg/ml. All spectra were corrected for the buffer contributions and the raw data output in ellipticity (θ), measured in millidegrees (mdeg) was converted to mean residue ellipticity (MRE, i.e., [θ] _MRW_) in deg cm^2^/dmol using the instrument software, Spectrum Manager 2. The CDPro package was used for secondary structure estimation using the programs SELCON, CONTINLL, and CDSSTR. The programs gave results that were similar to each other and relatively independent of the reference protein set used. The secondary structure calculations reported here were from processed CD spectra using the CDSSTR algorithm and the SP37A reference set.

### Crystallization, data collection, and structure determination

#### Cry6Aa trypsin-resistant core

The Cry6Aa trypsin-resistant core was concentrated to 100 mg/ml. Initial crystals were obtained using Rigaku Reagents, Inc. Wizard Classic I (Bainbridge Island, WA, USA). After screening multiple conditions, suitable crystals were obtained under kit condition 39 [20 % (w/v) PEG 1000, 0.1 M sodium phosphate dibasic/citric acid, pH 4.2; 0.2 M lithium sulfate]. Data were collected at 100 K from a single crystal on a Mar CCD-300 detector at LS-CAT (Advanced Proton Sources, Argonne National Laboratory). Data were indexed and processed with the HKL-2000 software suite [25].

The structure of this truncated form of Cry6Aa toxin was solved by the molecular replacement method using PHASER [26] (CCP4 package [27]) followed by manual rebuilding and model refinement. The poly-alanine chain of the crystal structure of hemolysin B from *Bacillus cereus* [PDB: 2NRJ] consisting of residues 19–334 was used as a search model. The final structure of the complex was obtained by carrying out several cycles of refinement consisting of manual model building using COOT [28], followed by restrained refinement with REFMAC [29] using Translation, Libration and Screw-rotation (TLS) refinement and the reference and secondary structure restraints. The trypsin-cleaved Cry6Aa structure, solved at higher resolution, was used as the reference structure in this refinement. Crystallographic data and refinement statistics are given in Table 1 and the Cry6A trypsin-resistant core structure can be found at the Protein Data Bank [PDB: 5KUC].

**Table 1:** Data collection and refinement statistics

PDB code	Cry6Aa trypsin-cleaved 5KUC	Full-length Cry6Aa 5KUD
Data collection		
Wavelength (Å)	1.12675	0.9786
Resolution range (Å)	50.00–2.00 (2.07–2.00)^a^	50.00–2.70 (2.80–2.70)
Space group	P 65	P 21 21 2
Cell dimensions		
*a*, *b*, *c* (Å)	112.967, 112.967, 76.627	50.367, 71.735, 142.913
α, β, γ (°)	90.0, 90.0, 120.0	90.0, 90.0, 90.0
Total reflections	135,786	97,894
Unique reflections	37,186 (3343)	14,755 (1454)
Multiplicity	3.7 (2.3)	6.6 (7.1)
Completeness (%)	98.80 (89.40)	99.50 (100.00)
< I>/sigma(I)	33.41 (4.51)	10.98 (2.88)
R-merge^b^ (%)	6.1 (22.9)	14.6 (89.4)
Refinement		
Resolution range (Å)	30.01–2.00 (2.06–2.00)	29.14–2.70 (2.80–2.70)
R-work/R-free^c^ (%)	18.77 (25.10)/22.80 (27.98)	27.43 (33.62)/32.48 (43.45)
Number of non-hydrogen atoms^d^	3605	3027
protein	3183	3006
ligand	0	0
water	422	21
Protein residues	402	393
RMS, bonds (Å)	0.009	0.013
RMS, angles (°)	1.071	1.650
Ramachandran favored (%)	98.74	97.93
Ramachandran outliers (%)	0.25	0.26
Average B-factor	30.70	84.00

*PDB* Protein Data Bank, *RMS* Root Mean Square deviation from ideal values (crystallography)

^a^Statistics for the highest-resolution shell are shown in parentheses

^b^R_merge_ = 100Σ(h)Σ(i)|I(i)-<I>|/ Σ(h)Σ(i)I(i) where I(i) is the **i**th intensity measurement of reflection h, and <I> is the average intensity from multiple observations

^c^R_factor_ = Σ||**F**_obs_|-|**F**_calc_||/ Σ|**F**_obs_|. Where **F**_obs_ and **F**_calc_ are the structure factor amplitudes from the data and the model, respectively. To calculate R-free values, 5 % and 10 % reflections were used for Cry6Aa trypsin core and full-length Cry6Aa structures, respectively.

^d^Per asymmetric unit

#### Purification of full-length Cry6Aa

Full-length Cry6Aa protein was concentrated to 15 mg/ml using Amicon centrifugal filters with a 10 kDa molecular weight cut-off (Millipore, USA) in 10 mM HEPES buffer, pH 7.5 and 25 mM NaCl.

Initial crystallization screens were performed using commercially available Classics, Classics Lite, Classics II, PEGs, PEGs II, PhClear, and PACT screens (Qiagen) by the sitting drop method in 96-round-bottom-well crystallization plates (Greiner Bio-One, GmbH, Germany) using a Mosquito Robotic System (TTP LabTech, Hertfordshire, UK). Diffraction quality Cry6Aa protein crystals were grown at 291 K from sitting drops containing 3 μl of the protein sample and 1.5 μl of reservoir solution (0.1 M citric acid, pH 4.6, 4 % PEG 6000). SDS-PAGE analysis of protein samples obtained by dissolving the crystals in SDS-buffer did not reveal any degradation products and confirmed the presence of only full-length Cry6Aa protein in the crystals used for the data collection.

For data collection, crystals were harvested with 20 % (v/v) glycerol in the reservoir solution. Diffraction data were collected at 100 K from a single crystal on a Mar CCD-300 detector at LS-CAT (Advanced Proton Sources, Argonne National Laboratory). Data were indexed and processed with HKL-2000 [25]. The crystals belonged to orthorhombic space group P2_1_2_1_2 and contained one molecule of full-length Cry6Aa per asymmetric unit.

The structure of full-length Cry6Aa was solved by molecular replacement using the structure of the truncated form of Cry6Aa as a search model. The final structure of full-length Cry6Aa toxin was obtained by carrying out several cycles consisting of manual model building using COOT [28], followed by structure refinement with REFMAC [29]. Crystallographic statistics are given in Table 1. Coordinates have been deposited in the Protein Data Bank [PDB: 5KUD].

#### Molecular modeling

The Cry6Aa tertiary structure was modeled ab initio using the Rosetta software [30]. After entering the Cry6Aa sequence, the first stage of the web-based process returned a secondary structure prediction with options to continue full protein structure prediction either using de novo or database structure comparison methods. Both methods were followed to produce a selection of five possible structure outcomes from both de novo and database predictions (10 models in total).

#### Intact molecular weight analysis/charged state distribution

Intact mass analysis was performed on an Agilent 1200 HPLC/MSD TOF 1969A system using a Michrom desalting trap heated to 50 °C. Each sample was diluted to a concentration of 0.2 μg/μl in 10 mM CAPS pH 11 buffer. Samples were also analyzed after reduction with 50 mM TCEP for 10 min. Approximately 1 μg protein was injected on column. The sample was eluted using a gradient (10 % buffer D for 1 min, 10–60 % buffer D over 2 min, 60–98 % buffer D over 2 min, 10 % buffer D for 1 min), where buffer A is 0.1 % formic acid in water and buffer D is 70 % isopropanol, 20 % acetonitrile, 10 % water, and 0.1 % formic acid. The mass was calculated using the Mass Hunter Qualitative Analysis software and the maximum entropy de-convolution algorithm.

Changes in the observed charge state distribution (CSD) of proteins analyzed by liquid chromatography–mass spectrometry (LC-MS), as described above, were used as an indirect probe of the overall conformational flexibility of the protein under different conditions.

#### MALDI-TOF mass spectrometry and tandem mass spectrometry

Digestion of Cry6Aa with trypsin results in two fragment fractions, a large fragment and a small fraction containing two peptides. The small fragment sample was analyzed by matrix-assisted laser desorption/ionization - time of flight mass spectrometry (MALDI-TOF/TOF MS) to determine the peptide masses and was subsequently fragmented in the LIFT mode to confirm the sequence of the peptides. The MALDI-TOF MS and tandem mass spectrometry (MS/MS) were conducted with a Bruker UltraFlextreme mass spectrometer. The C-terminal peptide (CTP) sample was diluted with 0.2 % trifluoroacetic acid (TFA) at 1:1 (v/v) and desalted using a C-18 Ziptip (Millipore). The peptides were eluted with 60 % acetonitrile (ACN) in 0.1 % TFA and mixed with a 2,5-dihydroxybenzoic acid (DHB) matrix [15 mg/ml in ACN:H_2_O (50:50)]. After spotting 1 μl of the mix on a MALDI sample plate, the peptide mass was analyzed using reflection-positive mode and the peptide was fragmented using the LIFT mode. The instrument was calibrated with CM 2 (calibration mixture 2, Peptide Mass Standards Kit, Applied Biosystems Sciex, P/N P2-3143-00). The mass spectrum was collected and analyzed using flex analysis software. The sequence was verified using BioTools software (Bruker) and the MASCOT search engine (Matrix Science).

#### N-terminal sequencing

Amino terminal sequencing of proteins was performed on a Shimadzu Protein Sequencer (Model PPSQ-33A) using basic Edman degradation chemistry. The protein sample was separated with SDS-PAGE under reducing conditions, and then the proteins were blotted onto polyvinylidene difluoride (PVDF) membrane by liquid transfer. A standard mix of 20 phenylthiohydantoin-amino acids (Shimadzu, catalog# 013-08391) was run each time. The amino acid residues from each Edman degradation cycle were determined based on their retention times from the C-18 column compared to standards.

#### Nematode bioassay

*C. elegans* Bristol N2 strain wild-type nematodes (*C. elegans* Genetic Stock Center) were cultured using standard techniques including the use of *E. coli* strain OP50 (*C. elegans* Genetic Stock Center) as standard food source [31] on enriched nematode growth (ENG) plates [32] containing 100 μM IPTG and 50 μg/ml kanamycin (ENG-IK plates [32]). To measure intoxication by Cry6Aa, 30–40 fourth-staged larvae of *C. elegans* were seeded on ENG-IK plates with *E. coli* Shuffle T7 Express LysY cells (New England Biolabs) transformed with the pET28b empty vector (negative control) or pET28b with *cry6Aa* inserts (wild-type and mutant forms) and incubated at 25 °C for 24 h. Three replica plates were used for each strain. Three to five worms were randomly picked from each of the different treatment conditions for image collection. Worm growth was assessed by measuring the area of clearly separated, individual worms (worms overlapping on image collection were not measured), using Image J (version 1.46r) and MATLAB (version R2010a) software [32]. The experiment was independently repeated three times. Data were analyzed with JMP v. 11 using one-way ANOVA and Dunnett’s post-test using the vector as the control group.

#### In vivo *pore formation assay*

Pore formation was assessed microscopically using the method described by Los *et al*. [24]. *C. elegans* Bristol N2 strain nematodes were synchronized by hypochlorite treatment. L1 animals were seeded onto OP50 and grown at 20 °C until the L4 stage was reached. *E. coli* JM103 cultures (ATCC) transformed with the empty pQE9 vector or vectors for expression of Cry5Ba or Cry6Aa were grown overnight at 37 °C with shaking in LB medium containing ampicillin. These cultures were diluted 1:10 in fresh medium and grown at 37 °C with shaking for 1 h before addition of IPTG to 50 μM and further incubation at 30 °C for at least 3 h with shaking. After this induction of protein expression, 30 μl of these cells, concentrated to an optical density of 2, were spread onto 60 mm diameter ENG agar plates containing ampicillin and IPTG and allowed to grow overnight at 25 °C for use in assays. Worms were then washed off the OP50 plate, washed twice to remove bacteria, and pipetted onto agar plates spread with the transformed JM103. Plates were incubated for 2 h at 20 °C. Worms were then washed off the plates, washed twice in M9 medium to remove bacteria, and rotated end-over-end at room temperature in M9 medium containing 5 mg/ml 5-hydroxytryptamine (serotonin, Sigma) for 15 min to stimulate feeding. Propidium iodide was then added to the medium and worms were rotated for an additional 30–40 min. Worms were washed twice to remove excess dye and pipetted onto agar pads on microscope slides and imaged. Pictures were taken using a Spot Insight CCD camera and Spot software connected to an Olympus BX60 compound microscope with an UplanFl 40×/0.75NA objective. At least 30 worms were imaged for each condition, and the entire experiment was conducted three times. The percentages for all three experiments were combined and analyzed by one-way ANOVA followed by Dunnett’s multiple comparison test between each toxin and the no-toxin control, using GraphPad Prism.

## Results and discussion

### Initial analysis

The Cry6Aa protein is active in the gut of nematodes such as *C. elegans*, which is an acidic environment with a pH ranging from ~3.6 to 6.0 [33] or Coleoptera such as *Diabrotica virgifera virgifera* with pH ranging from 5.75 to 6.03 [34]. The Cry6Aa crystals produced in *B. thuringiensis* and purified by sucrose gradient centrifugation could be solubilized at pH 10.0; however, Cry6Aa derived from this source showed a tendency to form aggregates and to precipitate after solubilization at either pH 3.0 or 12.7, particularly if this solubilization was followed by neutralization. As a result, further analyses utilized recombinant Cry6Aa produced in *P. fluorescens*. The secondary structure content of the full-length Cry6Aa protein from this source was estimated through CD analysis. At pH 3.0, results indicated an alpha helical content of ~73–74 % (depending on the protein reference used) with a beta sheet content of ~2–3.5 %. At pH 8.0, helical content of ~70–72 % and beta sheet of ~1–2 % was indicated. Results at pH 11.0 were impacted by the absorbance of the CAPS buffer below 200 nm and, thus, are less reliable but suggest that the protein remains predominantly alpha helical at this pH. Overall, and considering the inherent margins of error in CD determinations, these results suggest that the protein has a stable secondary structure across a broad pH range of 3.0 to 11.0.

### The structure of the trypsin-resistant core

**Fig. 1:**
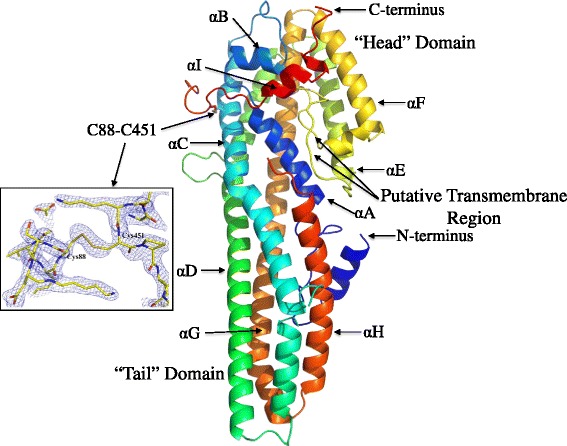
Crystal structure of Cry6a toxin. Ribbon representation of trypsin-truncated Cry6Aa form showing two domain architecture: the “tail” domain consists of one helical bundle with five long α-helices, labeled *αA*, *αC*, *αD*, *αG*, and *αH*, and shorter helices, labeled αB, αE, αF, and αI; while several long and short loops form the “head” domain. N- and C-termini and the putative transmembrane region are labeled. The Cys88-Cys451 disulfide bond is shown and, in the insert box, the final 2Fo-Fc electron density map calculated at 1.5σ in the region of this bond is shown in *blue mesh*. Side and main chains of the amino acid residues are presented as sticks and colored by the atoms

The structure of the trypsin-resistant core (Fig. 1) exhibits a group of long alpha helices (around 90 Å) that are amphipathic in nature, with their hydrophobic sides interacting and their hydrophilic faces showing greater exposure to solvent. This helix bundle is around 20–25 Å in diameter with a head domain folded across the helices at one end, formed from residues close to the C-terminus of the protein. The Cry6Aa trypsin-resistant core structure is unlike any previously described Cry protein although it is similar to several known toxins, including HlyE and HblB (see below).

Analysis of the trypsin-resistant core by SDS-PAGE following reduction and Edman degradation of the major band showed the N-terminal sequence HSLIH, corresponding to residues 12–16 of the Cry6Aa sequence and indicating processing after Arg11. Analysis by mass spectrometry of the trypsin-processed toxin, purified by ion exchange chromatography, without reduction, showed a dominant species with a mass of 46,844.19 Da and a minor species of 45,995.25 Da. The deconvoluted intact average molecular weight (MWavg) value of 46,844.19 Da matches the theoretical value (46,843.43 Da) expected for a core protein containing polypeptide chain 12–390 disulfide bonded to chain 444–475 with a mass error of +16.2 ppm. The minor species with MWavg of 45,995.25 matches the theoretical value of 45,996.51 for a core protein containing polypeptide chain 12–390 disulfide bonded to chain 451–475 with a mass error of +16.1 ppm. The mass errors for both of these measurements are within the expected mass accuracy of the LC-TOF instrument. The two CTPs, m/z 4114.894 and 3266.38 Da, were sequenced by MALDI MS/MS. This confirmed that the 4114.894 peak had the sequence NSNL*EYKC*PE*NNFMIYWYNN*SDWYNNSDWYN*N* (italicised residues were identified and assigned), which matched the CTP1 sequence of residues 444–475. The 3266.38 peak had the sequence CPE*NN*FMI*YWYNN*SD*WYNN*SDWY*NN*, which matched the CTP2 sequence from residues 451–475. These results show trypsin cleavage after Arg390 and processing sites after Arg443 and, at a lower frequency, after Lys450.

The solved structure of the trypsin-activated Cry6Aa corresponds to the major core of the toxin with the longer CTP1 fragment (from Asn444 to Trp472) still linked to the main body of the toxin by a disulfide bond. This bond can be clearly resolved in the structure (Fig. 1, insert). However, several residues were not resolved in the activated toxin structure: CTP1 residues Asn444 and Tyr473-Asn475; core region residues Lys126-Lys127 and Asn388-Ser389-Arg390, indicating that these regions are not fixed in the structure.

### The structure of full-length Cry6Aa

**Fig. 2:**
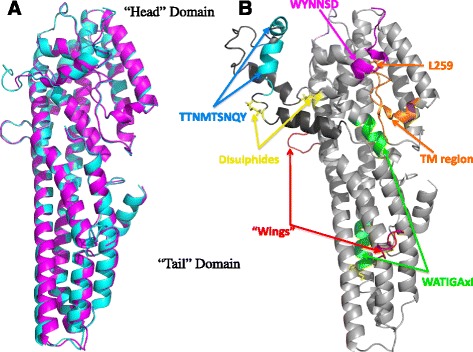
Comparison of Cry6Aa structures. **a** Superimposed ribbon representations of the crystal structures of the truncated (*cyan*) and the full-length (*magenta*) Cry6Aa forms. **b** The full-length Cry6Aa model is shown with the following features illustrated: wing-like intra-helical loops (*red*); putative transmembrane region (*orange*) with L259 shown in stick representation; WATIGAxI repeat sequence (*green*); TTNMTSNQY repeat sequence (*cyan*); WYNNSDWYNNSDW repeat (*magenta*); and the modeled Asn388–Lys450 in *dark gray* and *cyan*

The full-length structure of Cry6Aa [PBD: 5KUD] is slightly less structured than that of the trypsin-resistant core and was solved to a lower resolution (refinement statistics provided in Table 1). In the ordered regions where electron density can be traced, the structures are almost identical with a root mean square deviation (RMSD) for C-alpha atoms (total 391 matches after alignment) of 0.633 Å (Fig. 2a).

Mass spectrometric analysis of the full-length Cry6Aa used in crystallization revealed a deconvoluted molecular mass of 54,072.1 Da and, following reduction with dithiothreitol (DTT), a mass of 54,075.8 Da, corresponding to single full-length protein chain from Met1 to Asn475 and containing two disulfide bonds, as revealed by the mass difference of 4 between reduced and non-reduced states. The N-terminal 11 amino acids, the C-terminal six residues, Lys126–Lys127, and the region from Asn388 to Lys450 were not resolved, indicating significant flexibility in these regions. The latter region contains the trypsin cut sites described above and the flexibility of this region may contribute to its accessibility to this enzyme. The sequence from Cys451 to Asn469 is clearly seen in the structure and appears to be held in place by a disulfide bridge between Cys88 and Cys451 (see below).

To understand the activity of Cry6Aa from a structural point of view, and because some regions were unresolved in the structure, modeling was carried out to determine the conformation of internal missing segments and to build a full-length structure model. The Loop Modeler in MOE (Molecular Operating Environment, Chemical Computing Group, Montreal, Quebec, Canada) was used to find and select loop candidates using the PDB engine and to build model loops based on the desired loop sequence. After selecting a set of backbone loop candidates from the PDB loop library, optimization proceeded, including addition of side chains and a multi-stage energy minimization with pre-defined refinement protocols. The two regions Lys126–Lys127 and Asn388–Lys450 were modeled manually using COOT as described above to produce a structure-based model of residues 12–472. Ser386 and Tyr449 in the crystal structure were used as anchor residues and the region from residue 387 to 450 was used as the desired sequence to determine loop length and geometry restrictions. The loop searching calculation resulted in several model loops. The best model loop was chosen, based on its positioning of Cys88 and Cys451 in close proximity (consistent with the fact that they are disulfide-linked) and placing Cys402 close to Cys404, considering that the mass spectroscopy study indicated another disulfide bond. The two pairs of Cys residues were manually connected, followed by an energy minimization with MMFF94x force field. The resulting Cry6Aa model, from residue 12 to 472 (referred to below as the full-length model) has an alpha helical content of ~70 % and a beta sheet content of ~1.7 %, consistent with the CD data for this protein.

During the initial sequence analysis performed as part of this study, it was noted that the Cry6Aa sequence contains several internally repeated sequences that have not previously been noted. These can be visualized in the full-length model (Fig. 2b). The C-terminal domain contains two of these repeats: the sequence TTNMTSNQY (residues 406–414 and 418–426; cyan in Fig. 2b) and the repetitive sequence WYNNSDWYNNSDWYNN forms the extreme C-terminus of the protein (part of which is visible in the structure; magenta in Fig. 2b). The other repeat sequence, WATIGAxI(E/Q), occurs at residues 44–52 then at 332–340 and contributes to different alpha helices within the active core of the toxin (green in Fig. 2b).

### Disulfide bonding and conformational rigidity

The Cry6Aa sequence contains five Cys residues at positions 88 and 162 in the long alpha helical region, and at positions 402, 404, and 451 in the C-terminal region. The structures of the Cry6Aa trypsin-activated form and full-length Cry6Aa model show that Cys88 forms a disulfide bond with Cys451, while Cys402 appears to be joined to Cys404. Cys162 is not disulfide bonded in either full-length or activated Cry6Aa structures and it appears to face into the core of the alpha helical bundle where it is not surface exposed. The buried nature of this residue was confirmed by mass spectrometric analysis. Treatment of wild-type Cry6Aa under non-denaturing conditions with iodoacetamide resulted in no shift in deconvoluted intact average molecular weight, indicating that it is not accessible to modification. When the same protein was pre-treated with DTT followed by iodoacetamide, a mass change consistent with reduction of two disulfide bonds and alkylation of four cysteine residues was observed, suggesting that Cys162 remains inaccessible despite the overall increased structural flexibility caused by reduction of disulfide bonds.

**Fig. 3:**
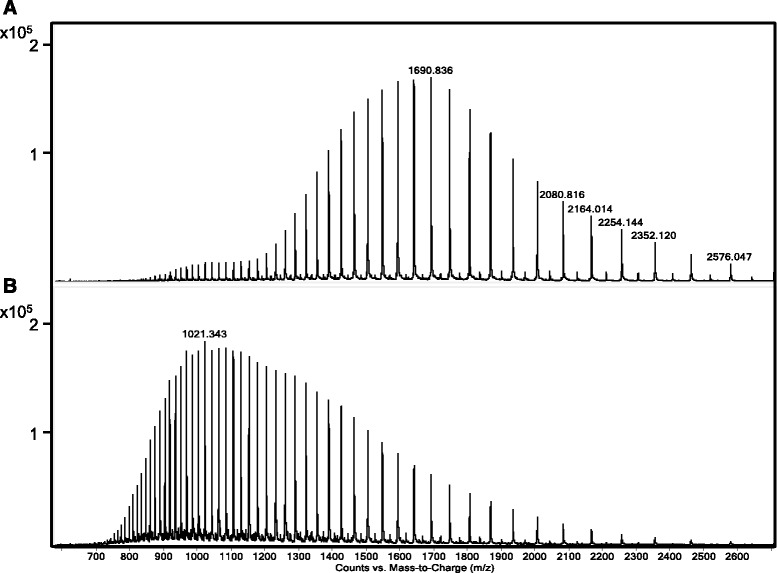
Charge state distributions (CSDs) of Cry6Aa. **a** Under non-reducing conditions the CSD centers around m/z 1690.84. **b** Following treatment with 1 mM dithiothreitol (DTT), the CSD centers around m/z 1021.34

The conformation of the Cry6Aa protein was also probed by mass spectrometry. Ionization of intact proteins under electrospray mass spectrometry conditions resulted in the detection of multiply charged species with characteristic CSDs unique for each protein. The distribution of charges (H^+^) on a protein under the denaturing conditions used for LC-MS is influenced by the primary sequence and the conformational flexibility of the protein, which determines the available protonation sites exposed on the denatured protein surface. This characteristic behavior of proteins is routinely used as an indirect probe of the flexibility of protein tertiary structure [35]. The role of the disulfide bonds in the overall rigidity of Cry6Aa was probed by measuring the CSD under different conditions (Fig. 3). Comparison of the CSD for Cry6Aa under non-reducing conditions and after treatment with 1 mM DTT shows a profound shift in the CSD (Fig. 3a and b), even though the deconvoluted intact average molecular weight only increased by +4. Under non-reducing conditions, the CSD is centered around m/z 1690.84, corresponding to a +32 charge state. After reduction, the CSD shifts to a center around m/z 1021.34, corresponding to a +53 charge state. This shift towards more highly charged species is a reflection of the decreased rigidity of the overall protein tertiary structure resulting from the reduction of the disulfides with DTT, exposing additional protein surfaces to protonation during ionization.

The pairing of cysteine residues and the buried nature of Cys162 described above indicate that natural Cry6Aa crystals are not stabilized by intermolecular disulfide bonds. This contrasts with many three-domain Cry protoxins in which disulfide bonds between the C-terminal proparts of individual protoxin molecules are important in crystal formation [36]. However, while Cry6Aa crystal stability does not appear to be mediated by covalent intermolecular bonding, we can speculate that the rigidity conferred on the Cry6Aa monomers by the intramolecular disulfide bridges could be relevant to crystal packing.

### Similarity to other toxins

It is interesting to note that in the related Cry6Ba sequence, the C-terminal domain found in Cry6Aa (88 amino acids shown in blue/cyan/magenta in Fig. 2b) is absent. As a result, the Cry6Ba sequence will lack both the C402–C404 and the C88–C451 disulfide bonds. There is no Cys residue at the position equivalent to Cys88 in a primary sequence alignment of Cry6Aa and Cry6Ba, where this residue is Asn94 (Additional file 1: Figure S1). Cry6Ba does, however, contain two additional cysteine residues at positions 100 and 125 in helix αC but these residues are too far apart to form an intramolecular disulfide bond. Cry6Ba also lacks two of the repetitive features described above (TTNMTSNQY repeat and C-terminal WYNNSD…) and the remaining repeat feature in Cry6Aa (WATIGAxIQ/E) is changed in both occurrences in Cry6Ba and becomes non-repetitive (WAFVQAYVT, residues 50-58, and WSVISLNIG, residues 340–348; Additional file 1: Figure S1). Cry6Ba has coleopteran activity but little or no nematode toxicity [20]. This indicates that this region is not necessary for toxicity per se. In the case of Cry6Aa, the C-terminal region is also not essential for toxicity since deletion of the C-terminal 93 residues and the first 10 residues from the N-terminus produces proteins that retain some, albeit reduced, toxicity against *C. elegans* [15]. The “wing” features seen in Cry6Aa (Fig. 1) are regions that vary in length when compared to the equivalent regions in Cry6Ba (Additional file 1: Figure S1). Since in Cry6Aa these features form interruptions to the αC and αD helices, we predict that these variations will be well tolerated in Cry6Ba.

**Fig. 4:**
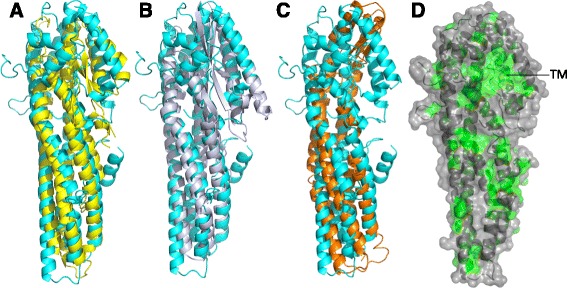
Comparison of Cry6Aa structure with structures of other related toxins. Superposition of Cry6Aa (*cyan*) with **a** HBL-B (*yellow*), **b** NheA (*gray*), and **c** HlyE (*orange*). In panel **d**, the surface representation of hydrophobic areas of the truncated Cry6Aa in ribbon representation are colored *green* and the remaining residues are colored *gray*. The putative transmembrane region is labeled (*TM*). C-terminal 463–472 residues are removed for simplicity

The structures derived for Cry6Aa are striking in their structural similarity to previously described bacterial toxins. A DALI search [37] conducted with the truncated Cry6Aa crystal structure identified the closest structural homolog as the HblB component of the *Bacillus cereus* hemolytic toxin [38] (pfam05791, [PDB:2NRJ]) with a significant Z-score value of 24.3 %, RMSD between 300 C∝-atoms of 2.8 Å and sequence identity of 12 % (Fig. 4a). The structural similarities to other toxins – such as the NheA component of the *B. cereus* non-hemolytic toxin [39] ([PDB: 4K1P], itself related to the NheC component of this toxin) and hemolysin E from *E. coli* [40] (HlyE, also known as ClyA or SheA [PDB: 4PHO]) – are also remarkable, with Z-scores of 21 % and 11 %, and RMSD values of 3.5 Å and 4.3 Å, despite low sequence identity of 12 % and 10 %, respectively (Figs. 4b and c). These are pore-forming toxins, which, like Cry6Aa, exhibit an elongated structure composed of an alpha helical bundle with a head domain at one end. Cry6Aa also shows some similarity to SMC_prok_A family proteins that are also known to form dimers [41]; in the related HlyE crystal structure, two monomers can be seen to pack together in a head-to-tail orientation [40]. Dimeric forms of Cry6Aa were observed in this study and have been described previously [14] but the packing of the monomers is not known. Despite this, there is no evidence for dimer formation in either crystal structure.

It is interesting to note that in Cry6Aa, two of the long alpha helices are interrupted with surface loops (shown in red in Fig. 2b) bounded by Gly residues (G_121_DPSIKKDG_129_ and G_189_DQKKLEG_196_):; the first of these loops is also seen in the ab initio models although the second was not predicted. These small, wing-like intra-helical loops are a unique feature of Cry6Aa not seen in the related structures of HlyE, HblB, or NheA and their significance is unknown at present. They do not appear to significantly interact with the rest of the molecule and residues Lys126 and Lys127 are not visible in the crystal structures, suggesting that they are mobile. Despite their apparent surface accessibility and the two lysine residues found near the tips of both of these loops, trypsin does not appear to cleave at these sites (described above). The HlyE mode of action model of Mueller et al. [42] and the recent work of Benke et al. [43] propose that upon interaction with the membrane, the putative pore-forming tongue region unfolds, causing the alpha helices to extend. It is possible that a similar rearrangement may occur for Cry6Aa, and that the G_121_DPSIKKDG_129_ and G_189_DQKKLEG_196_ loops reconfigure into alpha helical segments. This hypothesis is consistent with the observation that each loop is seven amino acids in length (two helical turns) and is bounded by glycine residues, which, due to the hydrogen R group, allow free rotation around the peptide bond, allowing a low energy secondary structure transition.

The head domain of Cry6Aa is folded across the helices as seen in the HblB [44] and NheA [39] structures (an orientation known to be somewhat distinct from that in HlyE [40]). Within the head domains of these proteins is a region termed the beta tongue that may contain the transmembrane sequence that allows them to form pores in target cells [38, 40, 44, 45]. In HlyE, this putative membrane-spanning in the beta tongue [46] is proposed to insert into the membrane along with the flanking helix E [45] to produce an oligomeric pore from either eight [47] or 13 [48] monomers. A similar hairpin loop feature, well conserved in Cry6Ba (Additional file 1: Figure S1) (residues 250–268, VEYSFLLGPLLGFVVYEIL) was identified both in the ab initio models and the structures of Cry6Aa (Fig. 4d and orange in Fig. 2b); analysis using the TMPRED software [49] predicts this to be a candidate transmembrane sequence.

### Probing the Cry6Aa mechanism of action

Prior to the completion of crystallographic analyses and the derivation of Cry6Aa structures, we initiated structural modeling of the Cry6Aa protein. Because the structural databases contained no proteins with primary sequence homology with Cry6, more detailed structural predictions based on known protein structures were impossible. We decided, therefore, to employ the Rosetta modeling program to predict the possible fold of the Cry6 protein ab initio, producing five de novo models with no reference to previous structures and five models using the database structure comparison method. All models (Additional file 2: Figure S2) exhibited a high proportion of alpha helix (44–56 %) and very low proportions of beta sheet (1.7–6.9 %). The modeling using the database structure comparison method returned a range of distinct structural models. In contrast, the five de novo models showed a high degree of similarity to each other, differing mainly in the disposition of the N- and C-terminal domains with respect to the central domain of the long alpha helices. These models predicted a predominantly alpha helical structure that is highly similar [the structural alignment of the backbone residues in the central region of the protein (residues 74–393], showing an RMSD of 1.78) to the actual structures of the protein that were subsequently solved through crystallography (see above). Based on the model, we were able to form a hypothesis that Cry6Aa is a pore-forming toxin and identify a region that might be involved in this function as a target for mutagenesis (see below).

### Nematode assays as a test for pore-forming activity of Cry6Aa

Given that our analyses showed structural homology to toxins such as HlyE and, by analogy, suggested a similar mechanism of action, we decided to test this hypothesis. In their work on HlyE, Wallace et al. [40] investigated the importance of the tongue region by substituting hydrophobic residues in this part of the sequence with non-hydrophobic amino acids. They demonstrated the loss of hemolytic activity. In a parallel experiment, to disrupt the hydrophobicity of the putative transmembrane region of Cry6Aa, we replaced Leu259 with Asp. The Leu259 residue was chosen because it is a hydrophobic residue at the center of the putative membrane-spanning domain (orange in Fig. 2b). The L259D-mutant protein was modeled and the change was not predicted to cause any alteration in the overall fold of the protein (Additional file 3: Figure S3). The wild-type Cry6Aa and the L259D mutant, both expressed in *E. coli*, were used in the bioassays against *C. elegans*.

**Fig. 5:**
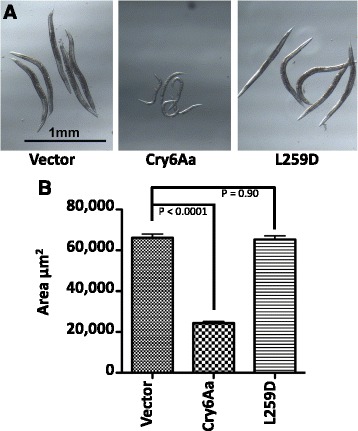
Nematode bioassay: *C. elegans* were fed on *E. coli* transformed with the pET28b plasmid as a negative control (*Vector*); transformed with this vector containing the wild-type *cry6Aa* gene (*Cry6Aa*); or transformed with the vector with the L259D mutant of this gene (*L259D*). **a** Images of some nematodes chosen at random are shown and **b** results are presented as mean worm area (12 worms per bar, each bar average from three independent experiments). *p* values comparing each condition to vector control are shown. Error bars represent the standard errors of the means

The expression of wild-type and mutant Cry6Aa in *E. coli* on IPTG induction was verified by western blotting (Additional file 4: Figure S4) and cells expressing these proteins were tested for their activity against *C. elegans*. Bioassays showed a striking (Fig. 5a) and significant (Fig. 5b) reduction in nematode growth following exposure to the wild-type Cry6Aa protein compared to those feeding on cells transformed with pET28b vector alone (Fig. 5). In contrast, the L259D mutant revealed no reduction in nematode size compared to the controls (Fig. 5a, b), indicating that this targeted mutation had eliminated Cry6Aa bioactivity against *C. elegans*. In combination with the structural data, this result is consistent with Cry6 acting as a pore-forming toxin, with structures and mechanisms of action similar to that of the HblB and HlyE toxins that could cause membrane damage through the insertion of residues into the head region of the protein, as postulated from the de novo models.

**Fig. 6:**
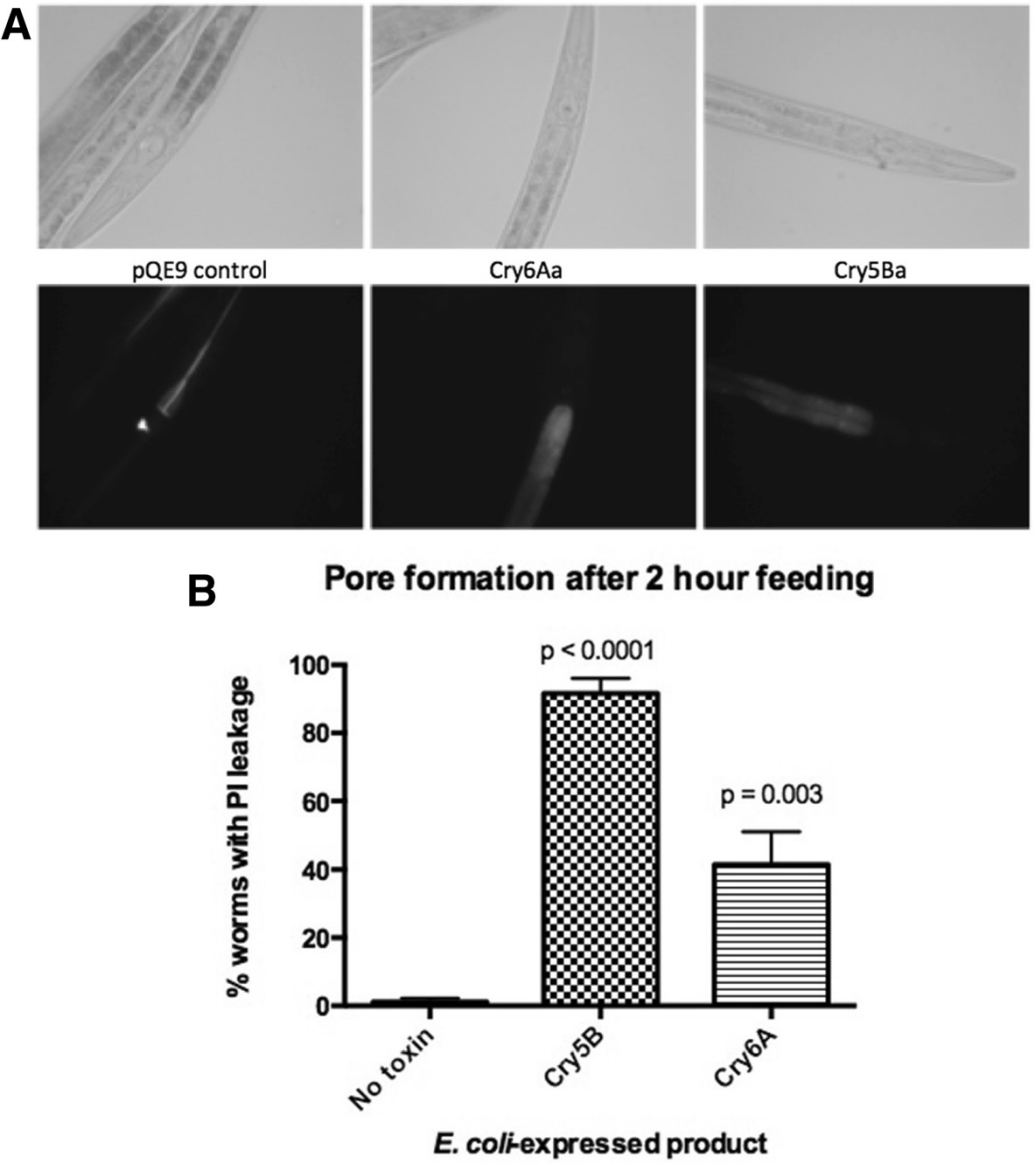
In vivo pore formation. **a** Bright field images (*upper panels*) and fluorescence images (*lower panels*) of the anterior regions of *C. elegans* fed on *E. coli* transformed with pQE9 vector or plasmids expressing Cry6Aa or Cry5Ba. **b** Percentage of treated worms showing propidium iodide (*PI*) uptake. The *p* values for the comparison between each toxin and the no-toxin control are shown

To test the ability of Cry6Aa to form pores more directly, experiments were conducted in vivo using *C. elegans* fourth-stage hermaphrodites and a live animal assay for the presence of pores in the intestine [24]. Following exposure of the hermaphrodites to *E. coli* either carrying empty pQE9 vector or expressing Cry6Aa or Cry5Ba, the penetration of ingested propidium iodide (PI) by intestinal cells was monitored. Under conditions used, PI gets into intestinal cells exposed to active pore-forming toxins [24]. As expected, nematodes fed with empty vector *E. coli* (pQE9, negative control, no pore-forming toxin) showed confinement of the PI to the lumen of the gut, whereas in Cry6Aa- and Cry5Ba (positive control [24])-treated hermaphrodites, entry of PI into the intestinal epithelial cell cytoplasm was evident (Fig. 6a). In these experiments the percentage of hermaphrodites showing PI entry was quantitated: for empty vector 1.1 ± 1.1 %, for Cry5B exposure 92 ± 4.4 %, and for Cry6Aa exposure 41.5 ± 9.7 %. The stronger effects of Cry5B on PI entry into intestinal cells could be due to factors such as the absolute toxicity of each protein, the relative numbers of receptors, or the relative quantities of the proteins expressed in the cells. Nonetheless, Cry6Aa showed significantly higher (*p* < 0.01 by ANOVA) pore formation than control (Fig. 6b). Therefore, based on all our data, we conclude that Cry6Aa is an active pore-forming toxin.

## Conclusions

**Fig. 7:**
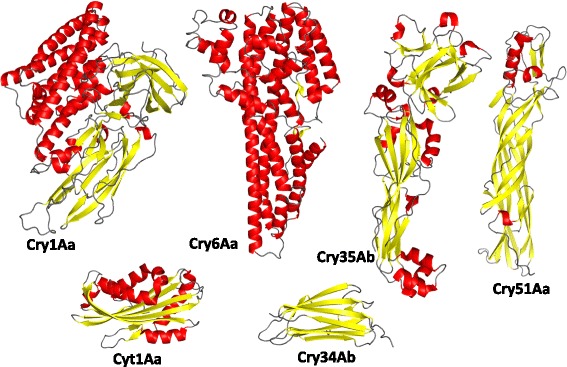
Structural families of *B. thuringiensis* delta-endotoxins. Representatives of different structural classes of delta-endotoxins of *B. thuringiensis* are shown. Cry1Aa [PDB: 1CIY] [52] is a three-domain toxin; Cry6Aa is an alpha helical toxin (this work); Cry34 is an aegerolysin-like protein [PDB: 4JOX] [12] that acts as a binary toxin with Cry35, a Toxin_10 family protein [PDB: 4JP0] [12]; Cry51 is a member of the Etx/Mtx2 family [PDB: 4PKM] [11]; and Cyt1Aa, a member of the Bac_thur_toxin family [PDB: 3RON] [53]. All structures are to scale and colored according to secondary structure (alpha helix, *red*; beta strands, *yellow*)

The elucidated crystal structures for Cry6Aa support a mechanism of action by pore formation, similar to toxins such as HblB, and we have demonstrated pore-forming ability in vivo. The HblB protein and the non-hemolytic toxin components NheB and NheC (which also show structural homology to HblB) are derived from *B. cereus* strains and induce pore formation and osmotic lysis of target cells [38], leading to the induction of diarrhea by this organism. *B. thuringiensis* is a member of the *B. cereus sensu lato* group of bacilli and, aside from production of invertebrate-active toxins by *B. thuringiensis*, the two species are indistinguishable by most tests [50, 51]. Interestingly, despite the low amino acid sequence identity, Cry6Aa shares a fold and, we propose, a mechanism of action with the *B. cereus* toxins and this may indicate an adaptation of this HblB protein family within the *B. cereus sensu lato* group for activity against target cells in invertebrates. The known structures of delta-endotoxins from *B. thuringiensis* (Cyt family; 3-domain Cry proteins; the aegerolysin-like Cry34 and Cry37; the Toxin_10 family Cry35; and the Etx/Mtx2 family Cry45, Cry46, and Cry51) are structurally distinct from the Cry6 fold (Fig. 7), which represents a new structural family among insecticidal toxins and a new paradigm for invertebrate pathogenic toxins. Based on the present study, we can now further elucidate key features of the Cry6Aa toxin that are responsible for its specificity and mode of action.

## Additional files

Additional file 1: Figure S1. Multiple sequence alignment of Cry6Aa and related toxins. Sequences of full-length Cry6Aa and Cry6Ba were aligned using the ClustalX program along with the sequences from the crystal structures of HlyE [PDB: 4PHO], HblB [PDB: 2NRJ], and NheA [PDB: 4K1P]. Residues identical to the Cry6Aa sequence are highlighted in green; residues identical to Cry6Ba but not in Cry6Aa are highlighted in blue. Repeat regions in Cry6Aa are highlighted in yellow (repeat motif 1 WATIGAxIQ/E, motif 2 TTNMTSNQY, and motif 3 WYNNSD). The putative transmembrane region is highlighted in gray with L259 indicated by a blue asterisk. The “wing” features in the Cry6Aa sequence are indicated by magenta highlight. (DOCX 19 kb) Additional file 2: Figure S2. Ab initio models of Cry6Aa. The five models constructed with reference to database information (db) and five models constructed entirely de novo (dn) are shown. The predicted N-terminal domains are shown in light gray and marked “N,” while the C-terminal domains are shown in black and marked “C.” (PPTX 774 kb) Additional file 3: Figure S3. Modeled L259D mutant. The full-length structure (5KUC, magenta) is overlaid with the L259D model (cyan). The inset illustrates the putative transmembrane loop region with residue 259 shown in stick display. Loops are colored as above for clarity while, for other regions, the full-length structure is dark gray and the modeled mutant is light gray. (PPTX 737 kb) Additional file 4: Figure S4. Western blot of wild-type and L259D mutant Cry6Aa. Expression of the wild-type Cry6Aa (WT) and the L259D mutant confirmed by western blotting. (PPTX 1138 kb)
