# Supplementary material for: The pesticidal Cry6Aa toxin from Bacillus thuringiensis is structurally similar to HlyE-family alpha pore-forming toxins
Source: BMC Biol. 2016 Aug 30;14(1):71. doi: 10.1186/s12915-016-0295-9 (PMC5004264; doi:10.1186/s12915-016-0295-9)
Supplement: Additional file 1: Figure S1. — Multiple sequence alignment of Cry6Aa and related toxins. Sequences of full-length Cry6Aa and Cry6Ba were aligned using the ClustalX program along with the sequences from the crystal structures of HlyE [PDB: 4PHO], HblB [PDB: 2NRJ], and NheA [PDB: 4K1P]. Residues identical to the Cry6Aa sequence are highlighted in green; residues identical to Cry6Ba but not in Cry6Aa are highlighted in blue. Repeat regions in Cry6Aa are highlighted in yellow (repeat motif 1 WATIGAxIQ/E, motif 2 TTNMTSNQY, and motif 3 WYNNSD). The putative transmembrane region is highlighted in gray with L259 indicated by a blue asterisk. The “wing” features in the Cry6Aa sequence are indicated by magenta highlight. (DOCX 19 kb) [file 12915_2016_295_MOESM1_ESM.docx]

**Figure S1**

|--1a---|

Cry6Aa MIIDSKTTLPRHS---LIHTIKLNSNKK---YGPGDMTNGNQFIISKQEWATIGAYIQTG

Cry6Ba1 MILGNGKTLPKHIRLAHIFATQNSSAKKDNPLGPEGMVTKDGFIISKEEWAFVQAYVTTG

HlyE -------------------TEIVCDKTVEVVKNAIETADGALDLYNKYLDQVIP------

HblB SLSEIEQTNNGDT------ALSANEARMKETLQKAGLFAKS---MNAYSYMLIKNPDVNF

NheA ---KEGQTEVKTV-------YAQNVIAPNTLSNSIRMLGSQSPLIQAYGLVILQQPDIK-

Cry6Aa LGLPVNEQQLRTHVNLSQDISIPSDFSQLYDVYCSDKTSAEWWNKNLYPLIIKSANDIAS

Cry6Ba1 TGLPINDDEMRRHVGLPSRIQIPDDFNQLYKVYNEDKHLCSWWNGFLFPLVLKTANDISA

HlyE ------------WQTFDETIKELSRFKQEYSQAAS------VLVGDIKTLLMDSQDKYFE

HblB EGITING-----YVDLPGRIVQDQKNARAHAVT---------WDTKVKKQLLDTLNGIVE

NheA -------------VNAMSSLTNHQKFAKANVRE---------WIDEYNPKLIDLNQEMMR

|-Wing-a-|

Cry6Aa YGFKVAGDPSIKKDGYFKKLQDELDNIVDNNSDDDAIAKAIKDFKARCGILIKEAKQYEE

Cry6Ba1 YGFKCAGKGATK--GYYEVMQDDVENISDNGYDKVAQEKAHKDLQARCKILIKEADQYKA

HlyE AT---------------QTVYEWAGVATQLLAAYILLFDEYNEKKAS-----AQKDILIK

HblB YDTTFDN--------YYETMVEAIN-----TGDGETLKEGITDLRG-------EIQQNQK

NheA YSIRFNS--------YYSKLYELAGNINEDEQSKADFTNAYGKLQLQVQSIQENMEQDLL

|--Wing-b----|

Cry6Aa AAKNIVTSLDQFLHGDQ----KKLEGVINIQKRLKEVQTALNQAHGESSPAHKELLEKVK

Cry6Ba1 AADDVSKHLNTFLKGGQDSDGNDVIGVEAVQVQLAQVKDNLDGLYGDKSPRHEELLKKVD

HlyE VLDDGITKLNEAQKSLL----VSSQSFNNASGKLLALDSQLTNDFSEKSSYFQSQVDKIR

HblB YAQQLIEELTKLRDSIG----HDVRAFGSNKELLQSILKNQGADVDADQKRLEEVLGSVN

NheA ELNRFKTVLDKDSNNLSIKADEAIKTLQGSSGDIVKLREDIKRIQGEIQAELTTILNRPQ

|--------*--------|

Cry6Aa NLKT----TLERTIKAEQDLEKKVEYSFLLGPLLGFVVYEILENTAVQHIKNQIDEIKKQ

Cry6Ba1 DLKK----ELEAAIKAENELEKKVKMSFALGPLLGFVVYEILELTAVKSIHKKVEALQAE

HlyE ----------KEAYAGAAAGVVAGPFGLIISYSIAAGVVEGKLIPELKNKLKSVQNFFTT

HblB YYKQ----LESD------------GFNVMKGAILGLPIIGGIIVGVARDNLGKLEPLLAE

NheA EIIKGSINIGKQVFTITNQTAQTKTIDFVSIGTLSNEIVNAADS-QTREAALRIQQKQKE

|--1b---|

Cry6Aa LDSAQHDLDRDVKIIGMLNSINTDIDNLYSQGQEAIKVFQKLQGIWATIGAQIENLRTTS

Cry6Ba1 LDTANDELDRDVKILGMMNSIDTDIDNMLEQGEQALVVFRKIAGIWSVISLNIGNLRETS

HlyE LSNTVKQANKDIDAAKLK--LTTEIAAIG-----EIKTETETTRFYCDYDDLMLSLLKEA

HblB LR---QTVDYKVTLNRVVGVAYSNINEMHKALDDAINALTYMSTQWHDLDSQYSGVLGHI

NheA LLPLIQKLSQTEAEATQITFVEDQVSSFTELIDRQITTLETLLTDWKVLNNNMIQIQKNV

Cry6Aa LQEVQDSDDA--DEIQIELEDASDAWLVVAQEARDFTLNAYSTNSRQNLPINVISDSCNC

Cry6Ba1 LKEIEEENDD--DALYIELGDAAGQWKEIAEEAQSFVLNAYTP

HlyE AKKMIN---------------TANEYQKRHGKKTLFEVPEV

HblB ENAAQKADQNKFKFLKPNLNAAKDSWKTLRTDAVTLKEGIKELKVETVTPQK

NheA EEGTYTDSSL----LQKHFNQIKKVSDEMNKQTNQFEDYVTNVEVH

|--2a---| |--2b---| |-3a-

Cry6Aa STTNMTSNQYSNPTTNMTSNQYMISHEYTSLPNNFMLSRNSNLEYKCPENNFMIYWYNNS

|| 3b-||-3c

Cry6Aa DWYNNSDWYNN
