# Supplementary figures and images for: The pesticidal Cry6Aa toxin from Bacillus thuringiensis is structurally similar to HlyE-family alpha pore-forming toxins
Source: BMC Biol. 2016 Aug 30;14(1):71. doi: 10.1186/s12915-016-0295-9 (PMC5004264; doi:10.1186/s12915-016-0295-9)

## Slide 1
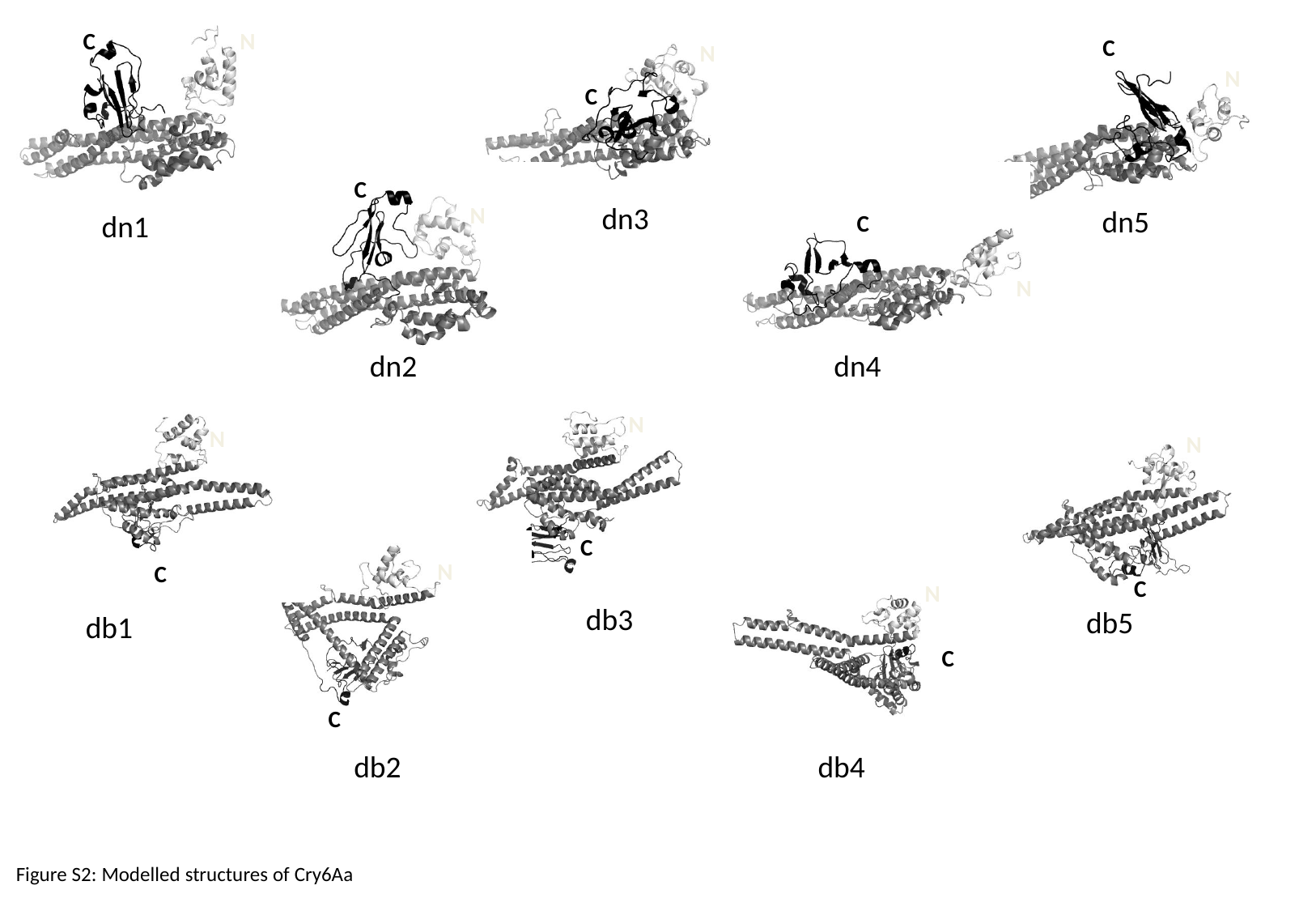

N
C
C
N
N
C
C
dn3
N
dn5
dn1
C
N
dn2
dn4
N
N
N
C
N
C
C
N
db3
db5
db1
C
C
db2
db4
Figure S2: Modelled structures of Cry6Aa

Supplement: Additional file 2: Figure S2. — Ab initio models of Cry6Aa. The five models constructed with reference to database information (db) and five models constructed entirely de novo (dn) are shown. The predicted N-terminal domains are shown in light gray and marked “N,” while the C-terminal domains are shown in black and marked “C.” (PPTX 774 kb) [file 12915_2016_295_MOESM2_ESM.pptx]

## Slide 1
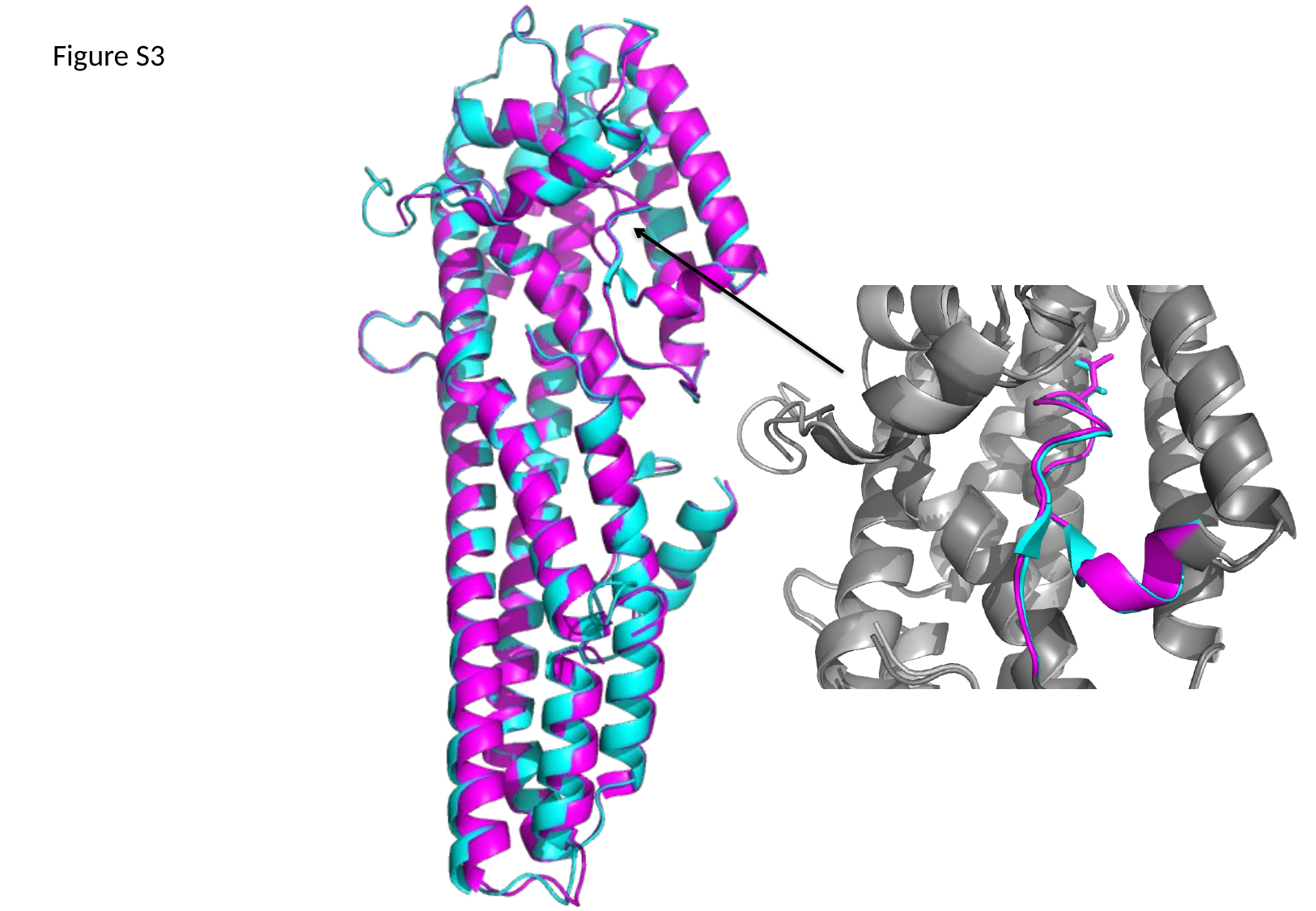

Figure S3

Supplement: Additional file 3: Figure S3. — Modeled L259D mutant. The full-length structure (5KUC, magenta) is overlaid with the L259D model (cyan). The inset illustrates the putative transmembrane loop region with residue 259 shown in stick display. Loops are colored as above for clarity while, for other regions, the full-length structure is dark gray and the modeled mutant is light gray. (PPTX 737 kb) [file 12915_2016_295_MOESM3_ESM.pptx]

## Slide 1
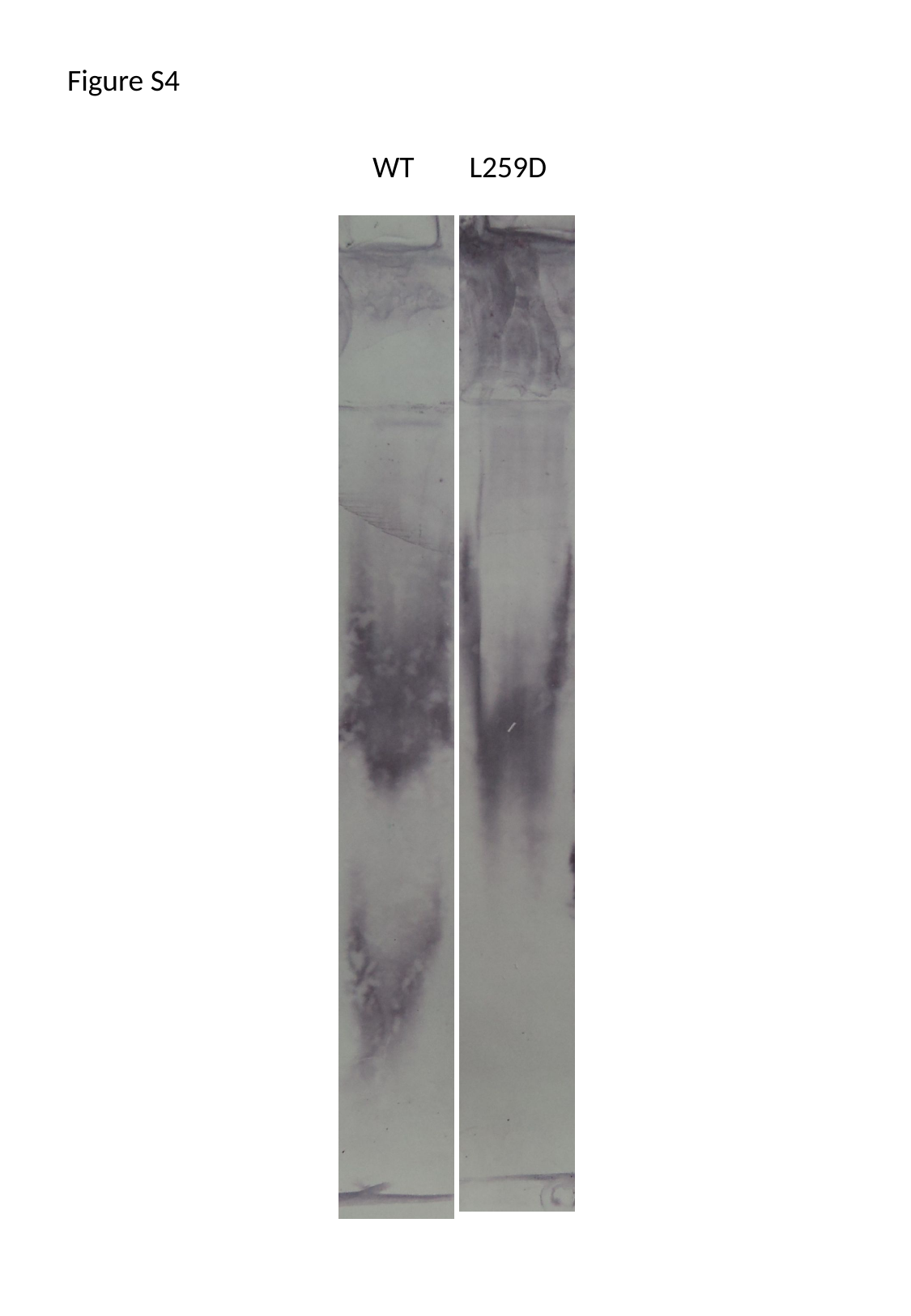

Figure S4
WT L259D

Supplement: Additional file 4: Figure S4. — Western blot of wild-type and L259D mutant Cry6Aa. Expression of the wild-type Cry6Aa (WT) and the L259D mutant confirmed by western blotting. (PPTX 1138 kb) [file 12915_2016_295_MOESM4_ESM.pptx]
